# Sonic Hedgehog Mediates the Proliferation and Recruitment of Transformed Mesenchymal Stem Cells to the Stomach

**DOI:** 10.1371/journal.pone.0075225

**Published:** 2013-09-19

**Authors:** Jessica M. Donnelly, Ambreesh Chawla, JeanMarie Houghton, Yana Zavros

**Affiliations:** 1 Department of Molecular and Cellular Physiology, University of Cincinnati College of Medicine, Cincinnati, Ohio, United States of America; 2 Department of Medicine, Division of Gastroenterology, and Department of Cancer Biology, University of Massachusetts Medical School, Worcester, Massachusetts, United States of America; University Hospital of Modena and Reggio Emilia, Italy

## Abstract

Studies using *Helicobacter*-infected mice show that bone marrow-derived mesenchymal stem cells (MSCs) can repopulate the gastric epithelium and promote gastric cancer progression. Within the tumor microenvironment of the stomach, pro-inflammatory cytokine interferon-gamma (IFNγ) and Sonic hedgehog (Shh) are elevated. IFNγ is implicated in tumor proliferation via activation of the Shh signaling pathway in various tissues but whether a similar mechanism exists in the stomach is unknown. We tested the hypothesis that IFNγ drives MSC proliferation and recruitment, a response mediated by Shh signaling. The current study uses transplantation of an *in vitro* transformed mesenchymal stem cell line (stMSC^vect^), that over-expresses hedgehog signaling, in comparison to non-transformed wild-type MSCs (wtMSCs), wtMSCs transfected to over-express Shh (wtMSC^Shh^), and stMSCs transduced with lentiviral constructs containing shRNA targeting the Shh gene (stMSC^ShhKO^). The effect of IFNγ on MSC proliferation was assessed by cell cycle analysis *in vitro* using cells treated with recombinant IFNγ (rmIFNγ) alone, or in combination with anti-Shh 5E1 antibody, and *in vivo* using mice transplanted with MSCs treated with PBS or rmIFNγ. *In vitro*, IFNγ significantly increased MSC proliferation, a response mediated by Shh that was blocked by 5E1 antibody. The MSC population collected from bone marrow of PBS- or IFNγ-treated mice showed that IFNγ significantly increased the percentage of all MSC cell lines in S phase, with the exception of the stMSCs^ShhKO^ cells. While the MSC cell lines with intact Shh expression were recruited to the gastric mucosa in response to IFNγ, stMSCs^ShhKO^ were not. Hedgehog signaling is required for MSC proliferation and recruitment to the stomach in response to IFNγ.

## Introduction

There is a clearly established role for Sonic Hedgehog (Shh) signaling in the development of cancer [1], [2], [3]. Cell growth in digestive tract tumors that include esophagus, stomach, biliary tract and pancreas are regulated by endogenous expression of Hedgehog ligands such as Shh [1]. Binding of Hedgehog ligand to its receptor, Patched (Ptch), results in removal of the inhibition of Ptch on Smoothened (Smo). This removal of the inhibition on Smo results in the activation of the Gli-family of Hedgehog transcription factors and subsequently tumor growth that can be blocked by Hedgehog neutralizing antibody [1]. While the association between Shh and gastric cancer is clear, the functional role of Shh in the development and progression of gastric cancer is largely unknown. Furthermore, the mechanism that regulates the production of Shh within the tumor microenvironment has yet to be determined.

*Helicobacter pylori (H. pylori)* bacterial colonization causes chronic inflammation that is consistently associated with the progression to gastric cancer [4]. The most common and detrimental immune response involves the Th1 pro-inflammatory cytokines, most prominently IFNγ from T cells, and IL-1β and TNFα from tissue or invading macrophages [5], [6], [7], [8], [9]. Indeed, pro-inflammatory cytokine IFNγ has been shown to contribute to the pathogenesis and development of gastric metaplasia [5], [9], [10] and cancer [10]. In inflammation-induced cancers, the Hedgehog signaling pathway mediates IFNγ-induced tumor development [11], [12], [13]. In particular, Shh is an IFNγ target gene and Hedgehog signaling a mediator of IFNγ-induced proliferation [12].

During *Helicobacter* infection, chronic inflammation coincides with the recruitment of bone marrow-derived mesenchymal stem cells (BM-MSCs) [14], [15]. In the chronically inflamed stomach BM-MSCs are recruited from bone marrow to the stomach and differentiate into cancer-associated fibroblasts (CAFs) that are instrumental in directing cancer development [14]. Although clearly implicated in the development of gastric cancer, the mechanism regulating the proliferation and recruitment of malignantly transformed BM-MSCs to the stomach during chronic inflammation is largely unknown. Interestingly, Shh is reported to induce proliferation and differentiation of BM-MSCs [16]. Shh has also been recognized as a potential chemoattractant for bone marrow derived cells when upregulated in response to chronic inflammation [17], [18].

Based on the association between IFNγ and Shh, we hypothesize that IFNγ induces Shh signaling within MSCs facilitating cell migration to the stomach. To test this hypothesis, the current study compares *in vivo* BM-MSC recruitment to the gastric mucosa in response to IFNγ using a spontaneously transformed mesenchymal stem cell line (stMSC) in comparison to untransformed BM-MSCs. In culture, BM-MSCs are prone to mutation with aging and exhibit clinically relevant mutations in the p53 gene [19]. With long-term culture BM-MSCs “spontaneously transform” (stMSCs), can be propagated in vitro for extended periods and exhibit a cancer-promoting phenotype [19]. The current study uses both stMSCs and untransformed BM-MSCs (wtMSCs) that over-expresses Hedgehog signaling. Using the wtMSC and stMSC cell lines both *in vivo* and *in vitro*, we demonstrate that IFNγ-induced proliferation and recruitment of MSC cell lines to the stomach is a response that is mediated by Shh secretion and signaling.

## Materials and Methods

### Animals

C57BL/6 (strain #000664) and IRG (strain #008705) mice used for these studies were purchased from Jackson Laboratories. All mouse studies were approved by the University of Cincinnati Institutional Animal Care and Use Committee (IACUC) that maintains an American Association of Assessment and Accreditation of Laboratory Animal Care (AAALAC) facility.

### Bone Marrow-derived Spontaneously Transformed Mesenchymal Stem Cells (stMSC) and Non-transformed BM-MSCs (wtMSC) Culture and Treatments

The wtMSC cell lines were established from whole bone marrow of IRG mice and cultured to passage 5 prior to transfection with subsequent maintenance at low passage number (<P10) prior to experimental use. Whole bone marrow was flushed from the femur and tibia of mice for subsequent culture and passage of the plastic adherent MSC population [20]. Spontaneously transformed bone marrow-derived mesenchymal stem cells (stMSCs) transfected with a pDsRed-Hyg-C1 plasmid expressing red fluorescent protein were used [21]. All cells were cultured using HyClone DMEM culture media supplemented with 10–15% fetal calf serum and 1% penicillin-streptomycin under normal conditions. MSCs cultured for treatment were plated in 6 well plates and allowed to grow to 70–80% confluence in normal media then serum starved overnight. After culture expansion, the Mouse Multipotent Mesenchymal Stromal Cell Marker Antibody Panel was used to label MSC cell lines for Sca-1, CD106, CD105, CD73, CD29, CD44 and the hematopoietic markers CD45 and CD11b (R&D Systems, SC018). Cells were suspended at a concentration of 1×10^6^ cells/ml and stained according to the manufacturer’s protocol. Cells were then incubated with AlexaFluor 488 at a 1∶100 dilution for 30 minutes at 4°C. Fixation was performed for 15 minutes at RT using a commercially available reagent (Invitrogen GAS001S5). Fluorescence intensity was measured by flow cytometry using the BD FACSCalibur and CellQuestPro software. wtMSCs and stMSCs were treated with vehicle, recombinant mouse interferon gamma (rmIFNγ, 100 nM, R&D Systems) or anti-Shh antibody 5E1 (pre-treated for 6 hours) plus rmIFNγ for 24 hours. Treated cells were then analyzed for changes in the cell cycle by flow cytometry.

### Sonic Hedgehog (Shh) Knockdown by Lentivirus-mediated Short Hairpin RNA (shRNA) using stMSCs

RFP-tagged stMSCs were transduced with five different MISSION lentiviral transduction particle clones containing different sequences of shRNA for Shh and a pLKO.1-puro component conferring puromycin resistance (Sigma Aldrich). RFP-tagged stMSCs were incubated with lentiviral particles with ExpressMag magnetic beads (Sigma Aldrich) on a magnet (Oz Biosciences Super Magnetic Plate, MF10000) for 15 minutes, cells were removed from the magnet, incubated for a further 16 hours and then placed under puromycin selection (DMEM Culture Media containing 10% fetal calf serum, 1% penicillin/streptomycin, 10 µg/mL puromycin) for 7 to 10 days. The effective puromycin dose needed to eliminate non-transduced cells was determined previously through a puromycin kill curve that tested concentrations from 0 to 50 µg/ml (data not shown). Transduced cells were denoted by a unique clone ID number with cell lines transduced with shRNA for Shh (stMSC^ShhKO^) numbered 59–63. A pLKO.1-puro vector transduced RFP-MSC cell line (stMSC^vect^) served as the control for the endogenous level of Shh gene expression. Western blot analysis was used to confirm Shh knockdown and RFP expression.

### Transfection of wtMSCs to Over-express Shh

The plasmid (Origene CW101340) was combined with the Origene Turbofectin reagent at ratios of 0∶1, 1∶1, 2∶1, 3∶1 and 4∶1 and incubated for 15 minutes at room temperature. The transfection reagent was then added drop-wise to wtMSCs at 70% confluence and allowed to incubate for 24 hours before cells were put under selection in normal media with puromycin. The effective puromycin dose needed to eliminate non-transfected cells was determined previously through a puromycin kill curve testing concentrations from 0 to 10 µg/ml (data not shown). The lowest concentration that killed all untransfected cells at 3–4 days post treatment was established as 2 µg/ml.

### In vivo stMSC Recruitment and Proliferation

C57BL/6 mice were transplanted with 1×10^6^ wtMSCs, wtMSCs^Shh^, stMSCs^vect^ or stMSCs^ShhKO^ via tail vein injection. Three days after transplantation, mice were injected with vehicle (sterile PBS, i.p.) or rmIFNγ (1 µg/day, i.p.) for 7 or 21 days. Mice were injected with 200 µl BrdU labeling stock reagent (5-Bromo-2′-deoxy-uridine Labeling and Detection Kit II, Roche Diagnostics, i.p.) 24 hours prior to analysis, and stomach sections fixed in 4% paraformaldehyde, paraffin-embedded and 3 µM sections prepared for immunohistochemical and immunofluorescence analyses. Whole bone marrow was also isolated from the femurs of the same mice with PBS flushing. Red blood cells were lysed using red blood cell lysis buffer (4.14 g NH_4_Cl, 0.5 g KHCO_3_, 0.5 M EDTA in 500 ml, pH 7.2–7,4). Approximately 11×10^6^ cells were fixed and permeabilized using Caltag Laboratories Flow Cytometry Kit, according to the manufacturer’s protocol (Invitrogen, GAS-004) and stained using anti-RFP FITC-conjugated antibody (1∶100, Abcam, ab34764) and then stained using Vybrant DyeCycle Ruby stain (Invitrogen) according to the manufacturer’s protocol. Fluorescence was analyzed using the BD LSRII Flow Cytometer system. To analyze specifically the RFP-MSCs, gating was used to analyze the cell cycle of only the FITC positive cells. Peak fluorescence was analyzed by ModFitLT software to determine the percentage of the population in each phase of the cell cycle.

### Flow Cytometry and Cell Cycle Analysis

Cells were serum-starved 16 hours prior to analysis. Cells were fixed with ice cold 70% Ethanol/PBS for 15 minutes at −20°C, washed and then stained with propidium iodide for 45 minutes. The measured values of peak fluorescence per total number of cells were obtained using the program CellQuest Pro (Becton Dickson). The percentage of cells from the population in each phase of the cell cycle was calculated from the peak fluorescence measurements through analysis with ModFit LT software. Flow cytometry using the FACSCalibur™ system (Becton Dickson) was performed on all transduced cells using the Mouse Multipotent Mesenchymal Stem Cell Marker Antibody Panel according to the manufacturers protocol (R&D Systems, SC018).

### Immunoprecipitation and Western Blot Analysis

Cell lysates were prepared using M-PER mammalian protein extraction reagent (Thermo Scientific, IL) supplemented with protease inhibitors (Roche) according to the manufacturer’s protocol. Media samples from cells were concentrated using 15 mL Amicon Ultra Centrifugal Filter Devices (Millipore). Cell lysates (100 µg total protein) and media were immunoprecipitated using anti-Shh 5E1 antibody (2 µg) at 4°C for 16 hours. Protein A/G agarose beads (20 µl, Santa Cruz Biotechnology, CA) were added and samples incubated at 4°C for 16 hours. After the 16 hour of incubation, immunoprecipitates were washed 3 times using PBS and resuspended in 40 µl Laemmli Loading Buffer containing β-mercaptoethanol (Bio-Rad Laboratories, CA) before loading onto 4–20% Tris-Glycine Gradient Gels. The gels were run at 80 V, 3.5 hours, protein transferred to nitrocellulose membranes at 105 V, 1–2 hours, and then blocked for 1 hour at room temperature using KPL Detector Block (Kirkegaard & Perry Laboratories, Inc.). Membranes were incubated overnight at 4°C with a 1∶200 dilution of Shh antibody (N-19, Santa Cruz) followed by a 1 hour incubation with 1∶100 dilution of AlexaFluor anti-goat 680 (Invitrogen). Blots were imaged using a scanning densitometer along with analysis software (Odyssey Infrared Imaging Software System).

### RNA Isolation and RT-PCR

Total RNA was isolated from stomach tissue using Trizol reagent according to the manufacturers protocol (TriReagent, Molecular Research Center, Inc.). The High Capacity cDNA Reverse Transcription Kit (Applied Biosystems) was used for cDNA synthesis from RNA following the recommended protocol. For each sample, 60 ng of RNA was reverse transcribed to yield approximately 2 µg total cDNA that was then used for RT-PCR. RFP primer sequences used were as follows: FORWARD –5′ CCC CGT AAT GCA GAA GAA GA 3′, REVERSE –5′ CTT GGC CAT GTA GGT GGT CT 3′. PCR amplifications were performed in a total volume of 20 µl, containing buffer, 20 mM forward and reverse primers, Taq polymerase, RNase-free water and cDNA template. Each PCR amplification was performed in duplicate wells in a GeneAmp PCR System 9700 thermocycler (Applied Biosystems), using the following conditions: 94°C 3 minutes, 94°C 30 seconds, 60°C 1 minute and 72°C 1 minute for 35 cycles. PCR products were visualized on a 1.5% agarose TAE gel.

### Immunohistochemistry

Mice were injected with 200 µl of BrdU labeling stock reagent (5-Bromo-2′-deoxy-uridine Labeling and Detection Kit II, Roche Diagnostics) 24 hours prior to analysis. Gastric tissues were fixed with Carnoy’s fixative (60 ml ethanol, 30 ml chloroform, 10 ml acetic acid) for 16 hours, paraffin embedded, and 4 µm sections were prepared. After deparaffinization, antigen retrieval was performed by heating the slides for 10 minutes at 100°C in 0.01 M sodium citrate buffer (Antigen Unmasking Solution, Vector Laboratories, Burlingame, CA). Endogenous peroxidase activity was then blocked by incubating slides in 3% hydrogen peroxide/ethanol for an additional 20 minutes. Sections were then blocked using 5% BSA/Tris buffered saline/0.1%Tween 80 (TBS-T) and incubated with a 1∶20 dilution of anti-BrdU antibody (5-Bromo-2′-deoxy-uridine Labeling and Detection Kit II, Roche Diagnostics) at 37°C for 30 minutes. BrdU color development was performed according to manufacturer’s protocol. Sections were then blocked with 20% normal goat serum for 20 minutes and incubated with a 1∶400 dilution of biotin-conjugated anti-RFP antibody (Abcam, ab34771) for 16 hours at 4°C followed by 1∶500 dilution of anti-rabbit IgG for 30 minutes and then visualized with avidin-biotin complexes using the Vectastain Elite ABC Kit using diaminobenzidine (DAB) as the substrate (Vector Laboratories, Inc., Burlingame, CA). Slides were mounted using Permount.

For adipocyte induction, stMSCs were treated with HyClone DMEM containing10^−8^ M dexamethasone (Sigma, D4902) and 5 µg/ml insulin (Sigma, I6634). All cells were fixed using 4% paraformaldehyde for 20 minutes at RT. To detect differentiation, Oil Red O staining was performed by adding a 3.75% Oil Red O solution, incubating 5 minutes at RT, then washing in water before mounting on slides using Vectashield HardSet Mounting Medium. Images were viewed and captured under light microscopy using an Olympus BX60 with a Diagnostic Instruments “Spot” Camera.

### Immunofluorescence

stMSC^vect^ and stMSC^ShhKO^ cells were grown on coverslips until 70–80% confluency, fixed with 4% paraformaldehyde for 30 minutes at room temperature, permeabilized with PBS containing 0.5% TritonX-100 and blocked with 2% donkey serum for 1 hour at room temperature. Cells were then incubated with either a 1∶50 dilution of anti-CD44 (Abcam ab65829) or a 1∶100 dilution of anti-CD45 (Abcam ab10558) antibody overnight at 4°C. Alexa Fluor donkey anti-rabbit 488 was used as the secondary antibody at 1∶1000 dilution for 1 hour at room temperature. Cells were then counterstained with a 1∶2000 dilution of the nuclear stain TO-PRO 3-iodide (Invitrogen, T3605) for 20 minutes at RT before mounting using Vectashield HardSet Mounting Medium. Rabbit IgG was used at a 1∶25 dilution as a negative control under the same conditions.

Stomach sections collected from PBS or IFNγ treated mice were blocked with 20% normal goat serum and incubated with 1∶400 anti-RFP antibody (Abcam, ab34771) for 16 hours at 4°C followed by 1∶100 anti-rabbit Alexa Fluor 488 (Invitrogen) secondary antibody for a further 1 hour. Slides were then counterstained using anti-E-cadherin (BD Transduction Labs, 610181) antibody at a 1∶200 dilution for 1 hour at room temperature followed by 1∶100 anti-mouse Alexa Fluor 633 (Invitrogen) secondary antibody. All samples were mounted using Vectashield Hardset Mounting Medium (VectaLabs). Images were obtained using a Zeiss LSM510 META confocal microscope.

### Chemotaxis Assay

The chemotaxis assay was performed based on established protocols [22]. Transduced stMSCs were plated in 24 well transwell plates containing PET membranes with 8 µm pores (BD Falcon). 2.5×10^4^ cells were seeded in the apical (upper) chamber in DMEM media containing 0.1% BSA. SDF-1α (rmSDF-1α, R&D Systems) was added to the basolateral (lower) chamber at concentrations of 0 to 150 ng/ml. Cells were incubated for 6 hours at 37°C. After migration, cells were fixed in 4% paraformaldehyde for 30 minutes then stained for 10 minutes using a Wright-Giemsa stain. Cells remaining on the top of the membrane were removed with a cotton-tipped applicator and remaining cells were counted in 5 fields at 40× magnification. The number of migrating cells was calculated by dividing the average number of migrating cells in each well by the average number of migrating cells in the control well.

### Statistical Analysis

Results were analyzed by either a student’s unpaired t test or ANOVA using commercially available software (GraphPad Prism, GraphPad Software, San Diego, CA). A *P* value <0.05 was considered significant.

## Results

### IFNγ-induced stMSC Proliferation is Mediated by Shh Secretion and Signaling

**Figure 1:**
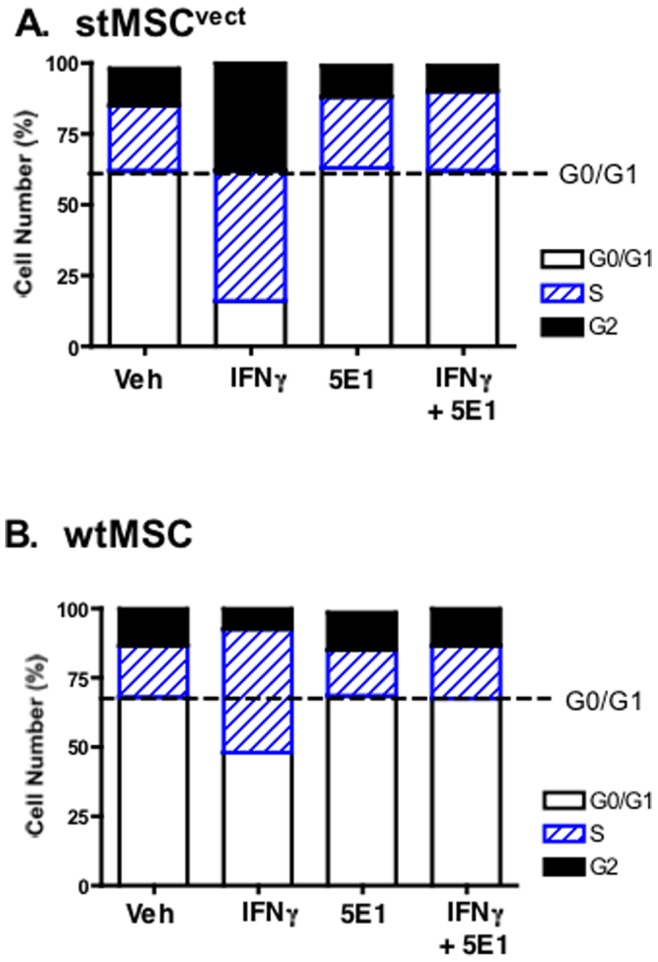
Effect of IFNγ on wtMSC and stMSC cycle progression. Cells were stained with propidium iodide and cell cycle analyzed by flow cytometry. The data were calculated from the cell cycle phase analysis by MODFIT software showing changes in distribution of G0/G1, S and G2/M phases from (**A**) stMSC- or (**B**) wtMSC-treated with vehicle (Veh), IFNγ, anti-Shh 5E1 antibody (5E1) and 5E1 plus IFNγ. Mean values from 4 individual experiments are shown.

The role of Hedgehog signaling as a mediator of IFNγ-induced stMSC and wtMSC proliferation was identified by flow cytometry and cell cycle progression. After treatment, cells were stained with propidium iodide and cell cycle analyzed by flow cytometry on a selected single cell population (**Figure S1A**). **Figure 1** represents the mean values of cell distribution percentage at different cell cycle phases in the treatment groups shown in **Figures S1B–E**. **Figures S1B–E** represent plots showing the changes in the cell cycle phases. The mean values and statistical significance in response to all treatments is summarized in **Figure S1F and G**. Analysis of the raw fluorescence data showed an increased percentage of stMSCs in S phase and G_2_/M phase, and a reduced percentage of cells in G_0_/G_1_ phase in response to rmIFNγ compared to the vehicle-treated cells (**Figure 1A**). To identify the role of secreted Shh as a mediator of IFNγ-induced stMSC proliferation we used anti-Hedgehog neutralizing 5E1 monoclonal antibody which binds to Shh ligand and disrupts protein binding to the receptor Ptch [1]. The proliferative response of stMSCs to rmIFNγ was significantly inhibited with 5E1 antibody treatment (**Figure 1A**). When compared to the stMSCs, non-transformed wtMSCs responded similarly in response to IFNγ suggesting the observed increase in proliferation was not the result of transformation alone. IFNγ significantly induced the percentage of wtMSCs in S phase, a response that was blocked by anti-Shh antibody (**Figure 1B**). Collectively, **Figure 1** demonstrates that IFNγ induces proliferation of both untransformed and spontaneously transformed MSCs, a response that is mediated by secreted Shh.

### Silencing the Shh Gene within Bone Marrow-derived stMSCs

To comprehensively study the role of Hedgehog signaling in BM-MSC proliferation and recruitment *in vivo*, wtMSCs were transfected to over-express Shh while stMSCs were transduced with lentiviral shRNAs against Shh. A pLKO.1-puro base lentiviral vector expressing a short hairpin sequence against the transcript of the Shh gene was used. Control MSC cell lines were established by using wtMSC with endogenous expression of Shh and transfection of stMSCs with the pLKO.1-puro base lentiviral vector (stMSC^vect^). Five lentiviral clones, each clone expressing a different short hairpin sequence, were analyzed (stMSC^ShhKO59^-stMSC^ShhKO63^). Knockdown of Shh was confirmed by Western blot analysis that showed 100% knockdown of Shh protein expression in all clones compared to the untransduced cells (wtMSCs) and Shh over-expressing cell lines (wtMSC^Shh^ and stMSC^vect^) (**Figure S2A**). Clone stMSC^ShhKO59^ was used for subsequent experiments and will be referred to as stMSC^ShhKO^. Expression of pDsRed-Hyg-C1 plasmid expressing red fluorescent protein (RFP) was confirmed by immunoblot using cell lysate collected from transduced stMSCs (**Figure S2A**). Importantly, wtMSCs stably transfected to over-express Shh protein, expressed Shh at a level similar to the stMSCs (**Figure S2A**).

To verify knockdown of Shh in MSCs without altering the differentiation of the cells, an expression pattern for the classical CD markers (CD29, CD106, CD105, CD44 and CD73) and Sca-1 using flow cytometry was performed using cultured stMSC^vect^ (**Figure S2B**) and stMSC^ShhKO^ (**Figure S2C**) cells. Both stMSC^vect^ and stMSC^ShhKO^ cells were negative for CD45 (**Figure S3**). Additionally, both stMSC cell lines were able to differentiate to an adipocyte phenotype based on positive Oil Red O staining of lipid droplets produced (**Figure S3**)**.** Collectively, these data suggest that knockdown of Shh protein in stMSCs did not change the differentiation of these cells.

### IFNγ Induces Proliferation of wtMSCs and stMSCs in Bone Marrow

**Figure 2:**
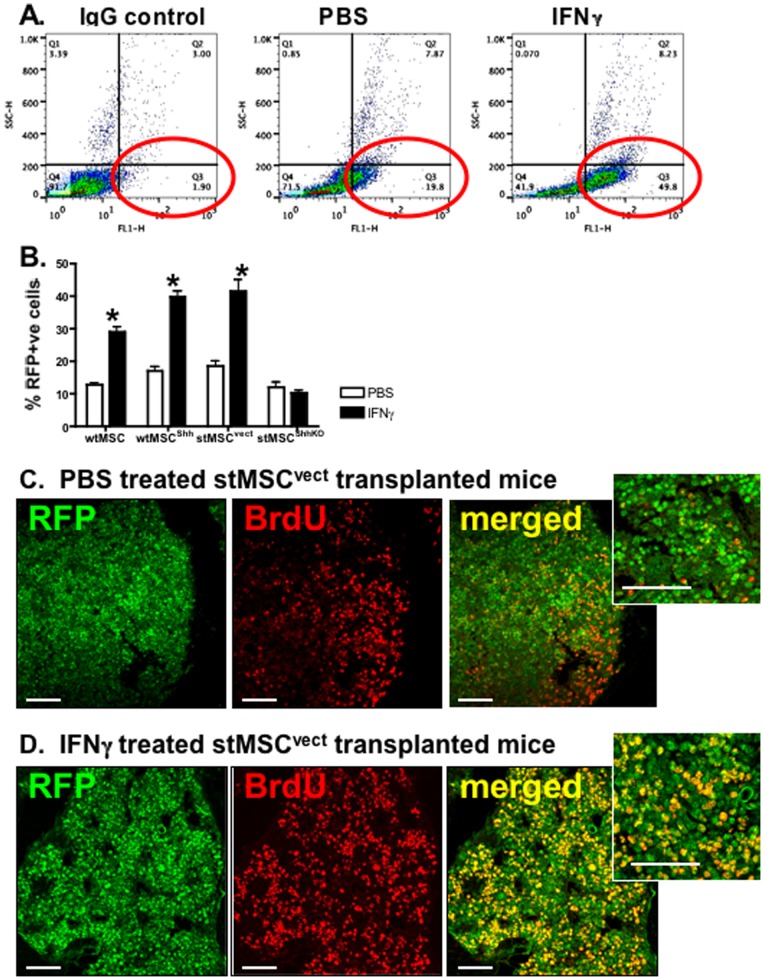
wtMSC and stMSC proliferation within the bone marrow compartment of mice injected with IFNγ. Flow cytometric analysis of bone marrow isolated from mice transplanted with wtMSC, wtMSC^Shh^, stMSCs^vect^ or stMSCs^ShhKO^ treated with rmIFNγ for 21 days. (**A**) Light-scatter analysis revealing an increase in RFP-positive MSCs in response to IFNγ. (**B**) Percentage of RFP-positive cells was quantified based on flow cytometric analysis. *P<0.05 compared to PBS-treated group, n = 4 mice/group. Representative immunofluorescence stain of RFP (green)- and BrdU (red)-positive cells within the bones collected from mice transplanted with stMSCs^vect^ treated with (**C**) PBS or (**D**) rmIFNγ for 21 days. Images captured at 10× magnification, insets captured at 40× magnification. Scale bar = 50 microns.

To determine the effect of IFNγ on wtMSC and stMSC proliferation *in vivo*, mice were transplanted with RFP-tagged wtMSC, wtMCS^Shh^, stMSC^vect^ or stMSC^ShhKO^ cells and treated with rmIFNγ for 21 days. After IFNγ treatment, bone marrow was harvested and analyzed for changes in cell cycle and number of RFP-positive cells by flow cytometry. IFNγ induced a significant increase in the number of RFP positive cells within the bone marrow of all experimental groups except for those animals transplanted with stMSC^ShhKO^ cells (**Figure 2A, B**). Immunofluorescence of bone marrow revealed that there was a significant increase specifically in the number of proliferating RFP positive cells in response to IFNγ as co-localized by BrdU immunostaining (**Figure 2C, D**). This was further supported by **Figure S4A, B and C** that shows representative light-scatter analysis revealing a population of stMSCs that were positive for RFP and comprised approximately 0.1% of the entire bone marrow cell population. RFP-positive cells were gated for the single cell population (**Figure S4C**) and cell cycle analyzed. Cells collected from the bone marrow of mice transplanted with stMSC^vect^ cells treated with rmIFNγ had a significantly greater percentage of cells in S-phase (55.5±2.82%) compared to those mice treated with PBS (32.9±5.14%, *P*<0.05 compared to stMSC^vect^ transplanted mice treated with PBS) (**Figure S4F**). The percentage of cells in S-phase collected from the bone marrow of mice transplanted with stMSC^ShhKO^ treated with rmIFNγ were not significantly different (32.9±2.64%) compared to those mice treated with PBS (38.4±1.47%, *P*>0.05 compared to stMSC^ShhKO^ transplanted mice treated with PBS) (**Figure S4I**). **Figures S4D, E, G and H** represent plots showing the changes in the cell cycle phases. Thus, IFNγ induces proliferation of both wtMSCs and stMSCs *in vivo* within the bone marrow, a response that appears to be mediated by Shh signaling.

### *In vivo* Recruitment of wtMSCs and stMSCs to the Gastric Mucosa in Response to IFNγ

**Figure 3:**
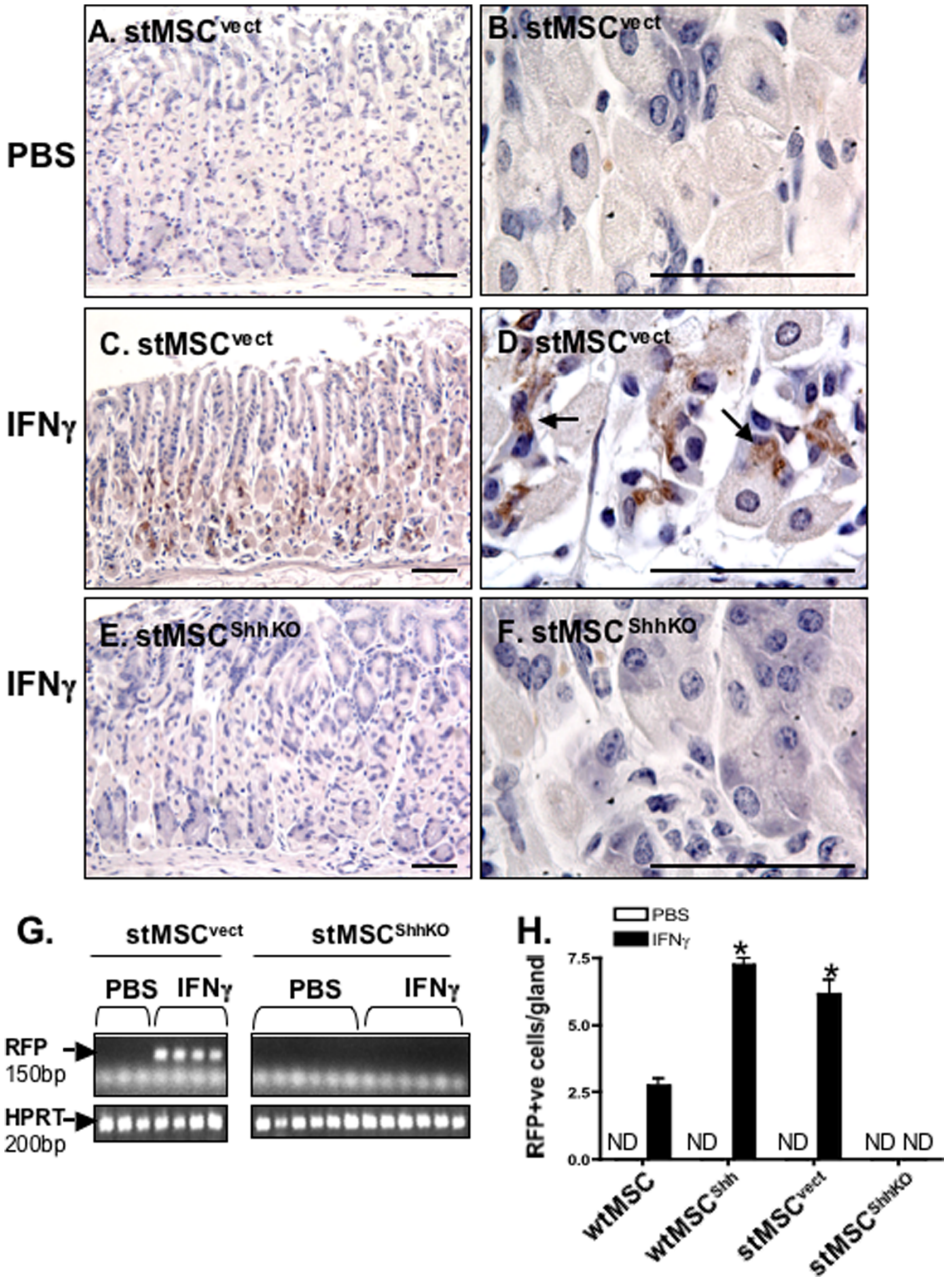
RFP-tagged stMSC^vect^ cells within the gastric mucosa of mice treated with IFNγ. Gastric mucosa collected from mice transplanted with stMSC^vect^ cells injected with (**A, B**) PBS or (**C, D**) IFNγ or with stMSC^ShhKO^ cells injected with (**E, F**) IFNγ were stained with anti-RFP antibody (brown). Higher magnification of image in (**A, C and E**) are shown in (**B, D and F**). (**G**) RT-PCR using RNA isolated from stomachs of PBS- or IFNγ-treated mice transplanted with stMSC^vect^ or stMSC^ShhKO^ cells. (**H**) Morphometric analysis of RFP-positive cells/gland in the stomachs of mice transplanted with wtMSC, wtMSC^Shh^, stMSCs^vect^ or stMSCs^ShhKO^ treated with rmIFNγ for 21 days. *P<0.05 compared to the wtMSC group, n = 4 mice/group. Images A, C and E captured at 20× magnification and images B, D and F captured at 100× magnification. Scale bar = 50 microns.

To identify the role of Hedgehog signaling as a mediator of IFNγ-induced stMSC recruitment to the gastric epithelium, mice transplanted with stMSC^vect^ and stMSC^ShhKO^ cells were treated with PBS or IFNγ for 21 days. Stomachs were then collected and recruitment of RFP-tagged stMSC^vect^ and stMSC^ShhKO^ cells analyzed by immunohistochemistry (**Figure 3**). stMSC^vect^ cells were recruited to the gastric mucosa of mice injected with IFNγ (**Figure 3C, D**) compared to the absence of RFP-tagged stMSC^vect^ cells in the stomachs of PBS-injected mice (**Figure 3A, B**). stMSC^ShhKO^ cells transplanted into mice injected with IFNγ were not recruited to the gastric mucosa (**Figure 3E, F**). Expression of RFP-tagged stMSC^vect^ cells with IFNγ treatment was confirmed by RT-PCR (**Figure 3G**). To identify whether recruitment of MSCs to the gastric mucosa in response to IFNγ was unique to the stMSCs, the experiment was repeated using either wtMSCs or wtMSCs over-expressing Shh (wtMSC^Shh^). Although both wtMSC and wtMSC^Shh^ cells were detected in the stomachs of IFNγ-treated mice, there were significantly higher numbers of wtMSC^Shh^ and stMSC^vect^ cells within the gastric mucosa when compared to the wtMSC cell transplanted group (**Figure 3H**). Collectively, these data suggest that Shh signaling is likely to mediate the recruitment of MSCs to the gastric mucosa in response to IFNγ.

**Figure 4:**
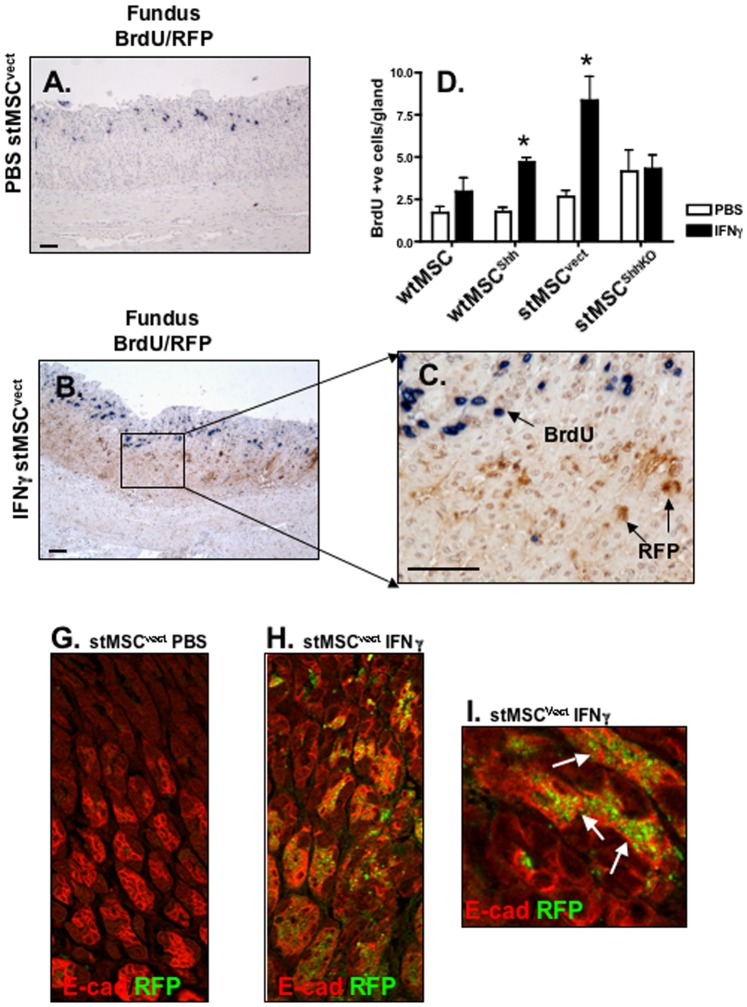
Proliferation of RFP-tagged stMSC^vect^ cells within the gastric mucosa. Gastric mucosa collected from mice transplanted with stMSC^vect^ cells injected with (**A**) PBS or (**B, C**) IFNγ were BrdU labeled (blue) and co-stained with anti-RFP antibody (brown). Higher magnification of image in (**B**) is shown in (**C**). (**D**) BrdU-labeled cells were quantified in the fundus. Cells were counted under 20× magnification. *P<0.05 compared to PBS-injected group, n = 4–6 mice/group. Confocal images of immunofluorescence staining for E-cadherin (E-cad, Alexa Fluor 633, red) and RFP (Alexa Fluor 488, green) mice transplanted with stMSC^vect^ cells injected with (**G**) PBS or (**H**) IFNγ. Higher magnification of image in (**H**) is shown in (**I**). Images A and B captured at 10× magnification and image C captured at 40× magnification. Scale bar = 50 microns.

There was a significant increase in the number of proliferating cells within the gastric mucosa in response to IFNγ-treated wtMSC^Shh^ and stMSC^vect^ transplanted mice compared to the PBS-injected mice (**Figure 4A–D** **, Figure S5A–F**). However, MSCs were BrdU negative (**Figure 4B, C** **and Figure S5A–D**). Although stMSC^ShhKO^ cells transplanted into mice injected with IFNγ were not recruited to the gastric mucosa, there was no difference in epithelial proliferation between PBS and IFNγ treatments in this experimental group, suggesting increased MSC-derived Shh may be required to support this increased proliferation (**Figure 4D**). In addition, although wtMSCs were recruited to the gastric mucosa in response to IFNγ, there was no difference in epithelial proliferation between the PBS and IFNγ treatments (**Figure 4D**). These data suggest that Shh signaling within wtMSCs^Shh^ and stMSCs in response to IFNγ induces proliferation within the gastric epithelium. IFNγ alone, in the absence of stMSCs, does not induce proliferation within the gastric mucosa confirming this effect is not related to inflammation alone.

To determine whether decreased stMSC proliferation, after recruitment, was associated with cell adhesion within the gastric epithelium, stomachs were then co-stained for E-cadherin and RFP (**Figure 4G–I**). PBS-treated mice that were transplanted with RFP-tagged stMSC^vect^ cells showed no recruitment of cells to the gastric mucosa (**Figure 4G**). Consistent with **Figure 3**, IFNγ-injected mice that were transplanted with RFP-tagged stMSC^vect^ cells showed marked recruitment of cells to the gastric mucosa (**Figure 4H**). RFP-tagged stMSC^Vect^ cells co-localized with E-cadherin (**Figure 4G, I**) supporting that recruited cells had engrafted within the gastric epithelium.

### Hedgehog Signaling Mediates the Expression of Focal Adhesion Protein in Response to SDF-1α Chemokine

**Figure 5:**
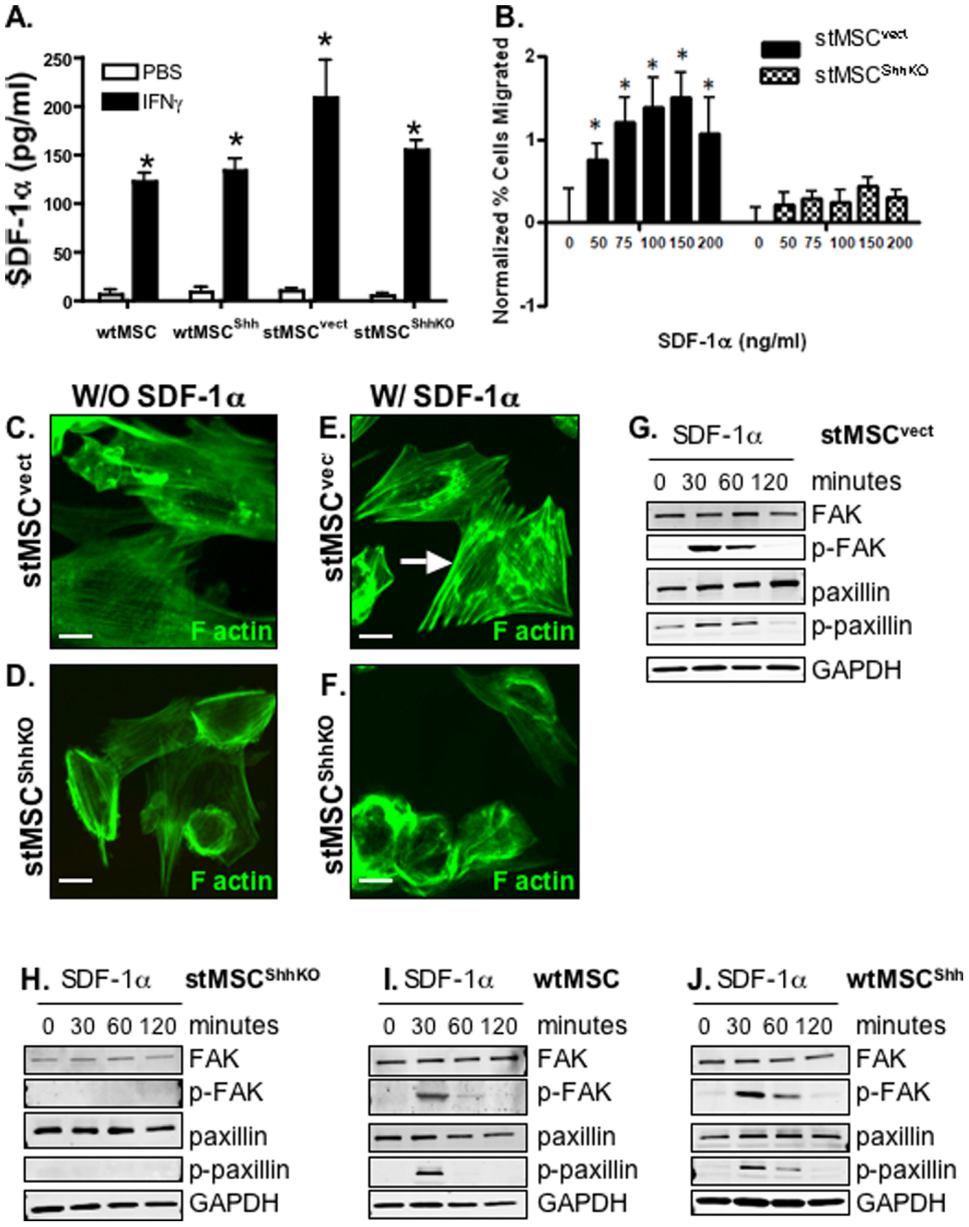
Focal adhesion kinase expression in wtMSCs and stMSCs in response to SDF-1α. (**A**) Circulating SDF-1α concentrations in plasma collected from mice transplanted with wtMSC, wtMSC^Shh^, stMSCs^vect^ or stMSCs^ShhKO^ treated with rmIFNγ for 21 days**.** (**B**) Migration assay using stMSC^vect^ and stMSC^ShhKO^ cells in response to chemokine SDF-1α at concentrations between 0 to 200 ng/ml. *P<0.05 compared to stMSC^ShhKO^ cells, n = 3 expts. F-actin re-organization in stMSC^vect^ and stMSC^ShhKO^ cells in response to SDF-1α. Immunofluorescence using an antibody specific for F-actin (Alexa Fluor 488, green) and nuclear marker (TO-PRO 633, red) of (**C, E**) stMSC^vect^ and (**D, F**) stMSC^ShhKO^ cells treated with SDF-1α. Arrows in **E** show actin stress fibers in stMSC^vect^ cells in response to SDF-1α. Immunoblots of FAK and paxillin protein expression in (**G**) stMSC^vect^, (**H**) stMSC^ShhKO^, (**I**) wtMSC and (**J**) wtMSC^Shh^ cells treated with SDF-1α. Images captured at 40× magnification. Scale bar = 10 microns.

We chose to investigate the SDF-1α/CXCR4 signaling axis as it has been established that this signaling pathway promotes MSC recruitment [23], [24]. There was a significant increase in circulating SDF-1α concentrations in all experimental groups in response to exogenous IFNγ (**Figure 5A**). Other cytokines such that included IL-11, IL-6, GM-CSF, IL-1β, TNFα and TGFβ were measured by Luminex® assay. We did not see any significant changes in these cytokines in response to exogenous IFNγ (data not shown). To begin to identify the mechanism by which Hedgehog signaling regulates MSC recruitment, we analyzed the migration of transduced stMSC^vect^ and stMSC^ShhKO^ cells in response to the chemokine SDF-1α (**Figure 5B**). While stMSC^vect^ cells migrated toward SDF-1α, transduced stMSC^ShhKO^ cells did not (**Figure 5B**).

Formation of actin stress fibers is a critical step for MSC migration [24]. Therefore we examined the effect of SDF-1α on cytoskeletal reorganization in stMSC^vect^ and stMSC^ShhKO^ cells. Actin stress fiber formation was observed in stMSC^vect^ cells stimulated with SDF-1α (**Figure 5C**) compared to the untreated stMSC^vect^ cells (**Figure 5D**). This organization was completely disrupted in stMSC^ShhKO^ cells treated with SDF-1α (**Figure 5F**) when compared to the untreated stMSC^ShhKO^ cells (**Figure 5E**). Disruption in the F-actin reorganization initiated by SDF-1α stimulation may explain the inability of stMSC^ShhKO^ cells to migrate in response to the SDF-1α gradient. Cell migration relies on the activation of tyrosine kinases such as focal adhesion kinase (FAK) and paxillin [24]. We observed a rapid transient increase in phosphorylated FAK and phosphorylated paxillin in all SDF-1α-treated cell lines except for stMSC^ShhKO^ cell (**Figure 5G–J**). This transient activation correlated with the re-organization of F-actin filaments shown in the representative **Figures 5C and D**. It is known that the SDF-1α/CXCR4 signaling axis is largely responsible for the homing of bone marrow-derived mesenchymal **[23]**, **[24]**. The loss of ability to migrate and activation of focal adhesion kinases that was associated with silencing of Shh expression suggests that Hedgehog is a key factor in this signaling cascade.

## Discussion

Sonic Hedgehog (Shh) secreted in response to IFNγ induced the proliferation of stMSCs within the bone marrow that may play a role in the malignant transformation of BM-MSCs as well as their tropism for sites of chronic inflammation. In culture, BM-MSCs are susceptible to p53 point mutations [19]. Normally BM-MSCs do not exhibit malignant properties, but with long-term culture BM-MSCs “spontaneously transform” (stMSCs) [19]. The stMSCs acquire cancer-promoting properties and interestingly have significantly increased growth kinetics [19], [25]. Moreover, in the chronically inflamed stomach BM-MSCs are recruited from bone marrow and contribute to a population of cancer-associated fibroblasts (CAFs) that are believed to drive cancer development [14]. Taken together with our findings demonstrating the rapid proliferation of both wtMSCs and stMSCs in response to IFNγ, we may speculate that during chronic inflammation IFNγ induces Shh secretion. Shh then acts via an autocrine regulatory mechanism to stimulate the rapid proliferation of MSCs within the bone marrow that could contribute to critical gene level changes and malignancy. These findings may have significance in aging and increased susceptibility to cancer development.

The expression of Shh protein within stMSCs was crucial for cell migration. In addition, there was a significant increase in circulating chemokine stromal cell-derived factor-1 (SDF-1α) concentrations in response to exogenous IFNγ in all experimental groups. Consistent with our observation, SDF-1α is known to promote the migration of human MSCs [24], [26]. With inflammation, the gastric mucosa exhibits increased levels of SDF-1 as a result of the production of hypoxia inducible factors and activation of the SDF-1/CXCR4 axis regulates the recruitment of stromal cells during the early stages of carcinogenesis [23], [27]. While discovering that MSCs are responsible for the development and progression of gastric carcinoma, the inflamed gastric mucosa exhibits increased levels of SDF-1α [15]. SDF-1α binds to its receptor CXCR4 to activate the Jak2/STAT3 and ERK1/2 signaling pathways that in turn regulate focal adhesion kinases (FAKs). Activation of FAKs subsequently induces reorganization of the actin cytoskeleton that promotes human MSCs migration [24]. There were marked differences between F-actin expression and activation of FAK and paxillin and the phosphorylated forms of these proteins between wtMSCs, wtMSC^Shh^ and stMSC^vect^ compared to the stMSC^ShhKO^ cells in our studies. While SDF-1α induced actin stress fibers and activation of FAK in wtMSC, wtMSC^Shh^ and stMSC^vect^ cells consistent with migration, stMSC^ShhKO^ cells exhibited a disorganized cytoskeleton, lack of phosphorylated FAK and paxillin in response to the chemokine, and failed to migrate to the gastric mucosa. Our data may suggest a role for Shh signaling in trafficking of the CXCR4 receptor to the membrane resulting in actin cytoskeletal reorganization leading to the migration of transformed MSCs from the bone marrow and recruitment to the stomach. It is well established that the CXCR4/SDF-1 pathway is implicated in the recruitment of MSCs to sites of injury as well as to areas of tumor development [24], [28], [29]. What emerges from this body of work is a plausible mechanism for the inflammation-initiated recruitment of MSCs to the stomach that is mediated by Hedgehog signaling.

Once recruited to the gastric mucosa in response to IFNγ, RFP-tagged wtMSCs and stMSCs were not actively proliferating but had adhered to the gastric epithelium, which was confirmed by the expression of E-cadherin. Consistent with published data, recruited stMSC^vect^ cells often appeared as single-scattered cells adhered to the gastric epithelium [15], [21], [30]. *In vitro* studies demonstrate that bone marrow-derived MSCs treated with gastric extract acquire an epithelial morphology that express the epithelial cell marker cytokeratin 19 and multiple gastric genes [30]. When injected into the adult stomach or early blastocysts MSCs give rise to gastric epithelial cells that may represent epithelial progenitor cells that are uncommitted to a particular cell lineage [30]. In the setting of chronic *Helicobacter* infection, bone marrow-derived cells repopulate the gastric mucosa and become engrafted within dysplastic glands and contribute to malignant progression [15]. Moreover, in mammary tissue under conditions of inflammation, MSCs acquire a cancer phenotype, while neoplastic dormancy was achieved with neutralization of the host’s inflammatory response [25]. Although exogenous IFNγ treatment causes gastritis and parietal cell atrophy [5], the duration of treatment (28 days) is not sufficient to cause dysplasia. Therefore, it was difficult from our current experiments to determine the role of MSCs within the stomach once recruited. However, our data does show that MSCs express and secrete Shh and that the Hedgehog signaling pathway is crucial for the recruitment to the stomach in response to IFNγ. In addition, the experimental groups that over-expressed Shh within MSCs (i.e. wtMSC^Shh^ and stMSC^vect^ cells) correlated with significantly increased epithelial proliferation once recruited to the gastric mucosa. Thus, the question that remains to be answered from the current and previous studies is the role of recruited MSCs within the stomach, whether untransformed or malignant.

With *H. pylori* infection T-lymphocytes recruited to the stomach produce IFNγ. During infection, IFNγ may trigger MSCs in the bone marrow to secrete elevated levels of Shh. Activation of this signaling pathway has a two-fold effect of not only increasing the proliferative capacity of MSCs but also increasing the ability to migrate in response to SDF-1α. However, once MSCs are recruited to the stomach, further studies are required to determine whether the inflammatory environment and Hedgehog signaling provide continual support for gastric carcinogenesis.

## Supporting Information

Figure S1 **Effect of IFNγ on stMSC cell cycle.** (**A**) Cells were stained with propidium iodide and cell cycle analyzed by flow cytometry on the single cell population. Flow cytometry graphs generated from cell cycle phase analysis by MODFitLT software showing changes in distribution of G0/G1, S and G2/M phases from stMSCs treated with (**B**) vehicle, (**C**) IFNγ, (**D**) immunoneutralizing anti-Shh 5E1 antibody (5E1) and (**E**) 5E1 plus IFNγ. The G_0_/G_1_ and G_2_/M phases are indicated as the two major peaks with arrows. The shaded blue area between the major peaks (G_0_/G_1_, G_2_/M phases) is the S phase. Cell cycle analysis by FACS showing changes in distribution of G0/G1, S and G2/M phases of all experimental groups that included **(F)** stMSCs and **(G)** wtMSCs treated with vehicle, IFNγ, anti-Shh 5E1 antibody and 5E1 plus IFNγ. *P<0.05 compared to vehicle-treated cells, n = 4 individual experiments.
(TIF)

Figure S2 **Transduction and transfection of MSCs.** (**A**) Western blot analysis demonstrating reduced expression of Shh in cultured, transduced stMSC clones compared to untransduced stMSCs^WT^. All available lentiviral constructs, each expressing a unique shRNA sequence targeting Shh gene expression, were assessed and were equally effective. They are denoted by the unique numbers within the clone ID numbers assigned by the manufacturer (ShhKO59-ShhKO63). Shh protein expression in media collected from cultured wtMSC, wtMSC^Shh^, stMSC^vect^ and stMSC^ShhKO^ cells confirming endogenous or over-expression of Shh. Flow cytometric analysis of MSC-specific cell surface markers for (**B**) stMSCs^vect^ and (**C**) stMSCs^ShhKO^.
(TIF)

Figure S3 **Differentiation of the transduced stMSC^vect^ and stMSC^ShhKO^ cell lines.** (**A**) stMSC^vect^ and (**B**) stMSC^ShhKO^ cells were stained with Oil Red O, with positive, red staining indicating lentiviral transduction does not affect stMSC ability to differentiate along normal cell lineages. stMSC^vect^ and stMSC^ShhKO^ cells were stained using IgG control (**C, D**), and were positive for the MSC marker CD44 (**E, F**) and negative for the hematopoietic stem cell marker CD45 (**G, H**).
(TIF)

Figure S4 **In vivo stMSC proliferation within the bone marrow compartment of mice injected with IFNγ.** Flow cytometric analysis of bone marrow isolated from mice transplanted with stMSCs^vect^ or stMSCs^ShhKO^ treated with rmIFNγ for 7 days. **(A, B)** Light-scatter analysis revealing a population of RFP-positive stMSCs. (**C**) RFP-positive cells were gated. Flow cytometric graphs generated from cell cycle phase analysis by MODFitLT software showing changes in distribution of G0/G1, S and G2/M phases from mice transplanted with stMSC^vect^ cells treated with (**D**) PBS and (**E**) IFNγ, or with stMSC^ShhKO^ cells treated with (**G**) PBS and (**H**) IFNγ. Graph generated from cell cycle phase analysis showing changes in distribution of G0/G1, S and G2/M phases from mice transplanted with (**F**) stMSC^vect^ or (**I**) stMSC^ShhKO^ cells treated with PBS and IFNγ. Data shown as mean ± SEM, n = 3–4 mice per group.
(TIF)

Figure S5 **Shh-expressing MSCs recruited to the gastric epithelium promote cell proliferation within the isthmus region.** Representative gastric sections isolated from mice transplanted with **(A, B)** wtMSC, **(C, D)** wtMSC^Shh^, stMSC^vect^ and **(E, F)** stMSC^ShhKO^ cells were stained using antibodies against BrdU (blue) and RFP (brown). RFP positive MSCs (brown) were recruited to the gastric mucosa in response to IFNγ in the **(B)** wtMSC and **(D)** wtMSC^Shh^ transplanted groups but this recruitment was lost in the **(F)** stMSC^ShhKO^ transplanted groups. In groups with MSC recruitment in response to IFNγ **(B, D)**, BrdU (blue) and RFP (brown) staining do not overlap, although the number of proliferating cells increase, suggesting recruited MSCs do not represent the proliferative population but instead promote gastric epithelial cell proliferation. Images captured at 10× magnification. Scale bar = 50 microns.
(TIF)
