# Supplementary figures and images for: Sonic Hedgehog Mediates the Proliferation and Recruitment of Transformed Mesenchymal Stem Cells to the Stomach
Source: PLoS One. 2013 Sep 19;8(9):e75225. doi: 10.1371/journal.pone.0075225 (PMC3777931; doi:10.1371/journal.pone.0075225)

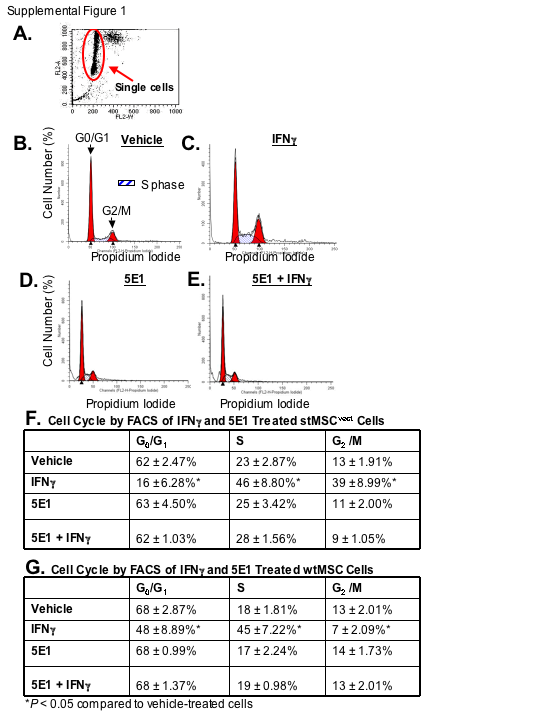

Supplement: Figure S1 — Effect of IFNγ on stMSC cell cycle. (A) Cells were stained with propidium iodide and cell cycle analyzed by flow cytometry on the single cell population. Flow cytometry graphs generated from cell cycle phase analysis by MODFitLT software showing changes in distribution of G0/G1, S and G2/M phases from stMSCs treated with (B) vehicle, (C) IFNγ, (D) immunoneutralizing anti-Shh 5E1 antibody (5E1) and (E) 5E1 plus IFNγ. The G0/G1 and G2/M phases are indicated as the two major peaks with arrows. The shaded blue area between the major peaks (G0/G1, G2/M phases) is the S phase. Cell cycle analysis by FACS showing changes in distribution of G0/G1, S and G2/M phases of all experimental groups that included (F) stMSCs and (G) wtMSCs treated with vehicle, IFNγ, anti-Shh 5E1 antibody and 5E1 plus IFNγ. *P<0.05 compared to vehicle-treated cells, n = 4 individual experiments. (TIF) [file pone.0075225.s001.tif]

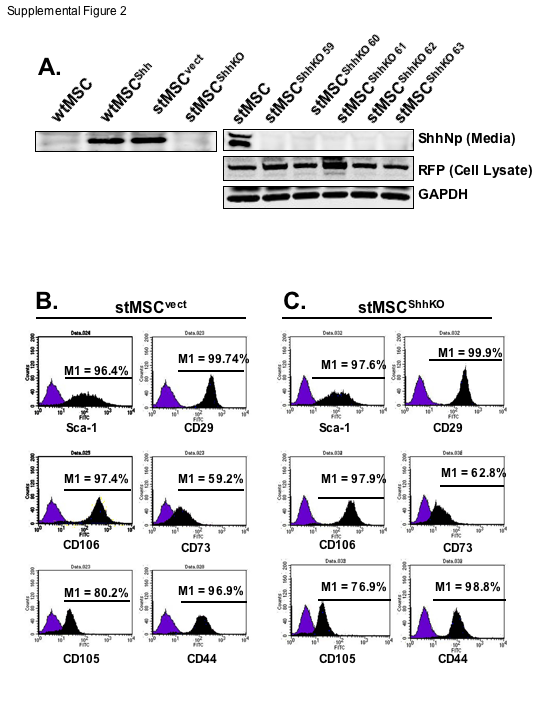

Supplement: Figure S2 — Transduction and transfection of MSCs. (A) Western blot analysis demonstrating reduced expression of Shh in cultured, transduced stMSC clones compared to untransduced stMSCsWT. All available lentiviral constructs, each expressing a unique shRNA sequence targeting Shh gene expression, were assessed and were equally effective. They are denoted by the unique numbers within the clone ID numbers assigned by the manufacturer (ShhKO59-ShhKO63). Shh protein expression in media collected from cultured wtMSC, wtMSCShh, stMSCvect and stMSCShhKO cells confirming endogenous or over-expression of Shh. Flow cytometric analysis of MSC-specific cell surface markers for (B) stMSCsvect and (C) stMSCsShhKO. (TIF) [file pone.0075225.s002.tif]

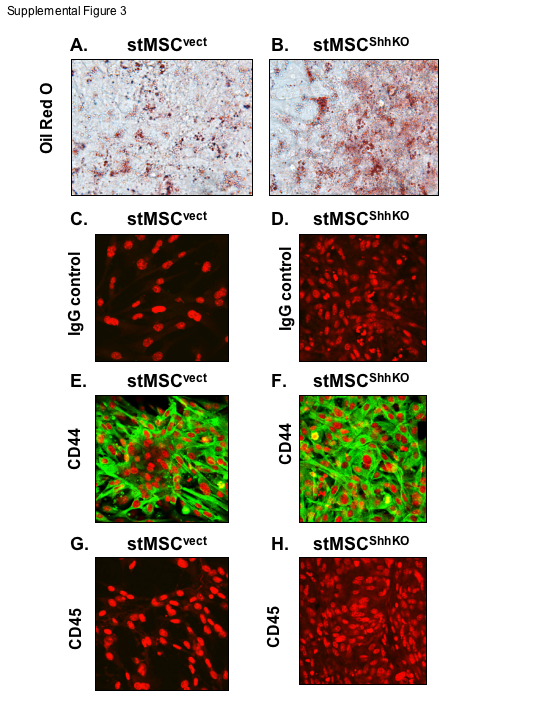

Supplement: Figure S3 — Differentiation of the transduced stMSCvect and stMSCShhKO cell lines. (A) stMSCvect and (B) stMSCShhKO cells were stained with Oil Red O, with positive, red staining indicating lentiviral transduction does not affect stMSC ability to differentiate along normal cell lineages. stMSCvect and stMSCShhKO cells were stained using IgG control (C, D), and were positive for the MSC marker CD44 (E, F) and negative for the hematopoietic stem cell marker CD45 (G, H). (TIF) [file pone.0075225.s003.tif]

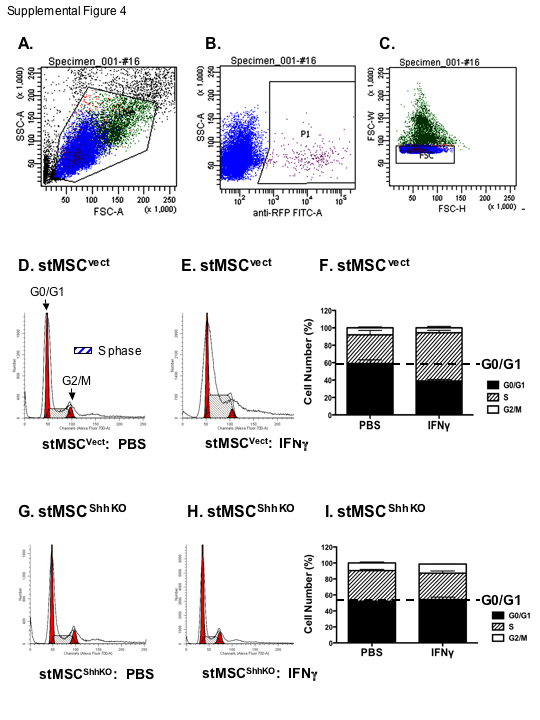

Supplement: Figure S4 — In vivo stMSC proliferation within the bone marrow compartment of mice injected with IFNγ. Flow cytometric analysis of bone marrow isolated from mice transplanted with stMSCsvect or stMSCsShhKO treated with rmIFNγ for 7 days. (A, B) Light-scatter analysis revealing a population of RFP-positive stMSCs. (C) RFP-positive cells were gated. Flow cytometric graphs generated from cell cycle phase analysis by MODFitLT software showing changes in distribution of G0/G1, S and G2/M phases from mice transplanted with stMSCvect cells treated with (D) PBS and (E) IFNγ, or with stMSCShhKO cells treated with (G) PBS and (H) IFNγ. Graph generated from cell cycle phase analysis showing changes in distribution of G0/G1, S and G2/M phases from mice transplanted with (F) stMSCvect or (I) stMSCShhKO cells treated with PBS and IFNγ. Data shown as mean ± SEM, n = 3–4 mice per group. (TIF) [file pone.0075225.s004.tif]

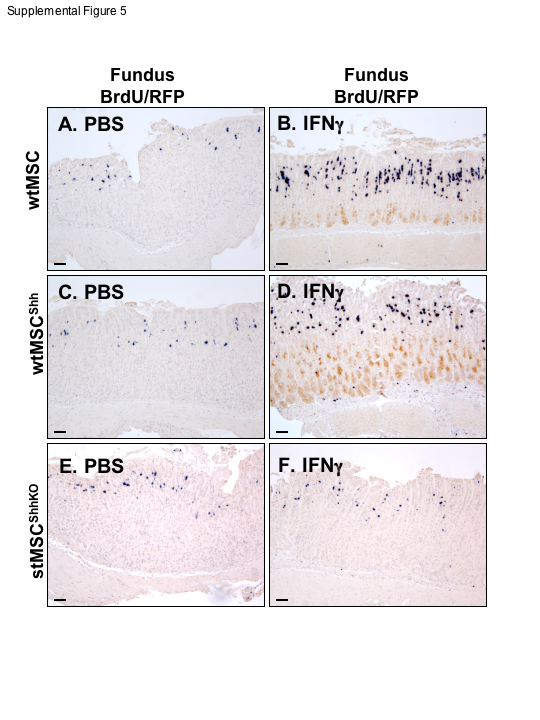

Supplement: Figure S5 — Shh-expressing MSCs recruited to the gastric epithelium promote cell proliferation within the isthmus region. Representative gastric sections isolated from mice transplanted with (A, B) wtMSC, (C, D) wtMSCShh, stMSCvect and (E, F) stMSCShhKO cells were stained using antibodies against BrdU (blue) and RFP (brown). RFP positive MSCs (brown) were recruited to the gastric mucosa in response to IFNγ in the (B) wtMSC and (D) wtMSCShh transplanted groups but this recruitment was lost in the (F) stMSCShhKO transplanted groups. In groups with MSC recruitment in response to IFNγ (B, D), BrdU (blue) and RFP (brown) staining do not overlap, although the number of proliferating cells increase, suggesting recruited MSCs do not represent the proliferative population but instead promote gastric epithelial cell proliferation. Images captured at 10× magnification. Scale bar = 50 microns. (TIF) [file pone.0075225.s005.tif]
